# Gender: the state of being male or female

**DOI:** 10.1007/s12471-015-0738-x

**Published:** 2015-07-22

**Authors:** E.E. van der Wall

**Affiliations:** Netherlands Society of Cardiology/Holland Heart House, Moreelsepark 1, 3511 Utrecht, EP The Netherlands

The American College of Cardiology (ACC) CV News Digest is a daily news briefing selected from thousands of media sources (including the Wall Street Journal and New York Times). The mission of CV News Digest is to alert ACC members about cardiovascular-related information that their patients may read or hear in the media that day or week. Being a fellow of the ACC one is almost daily informed about specific topics that have penetrated the news media. In 2010, I informed you about the gender issues that had been reported by AAC CV News Digest in the period from December 2009 till June 2010 [1]. Since gender issues continue to have increasing attention these days (cardiovascular disease in women is currently one of the five major strategic themes of the Netherlands Heart Foundation), I have repeated the search for news provided by ACC CV News Digest that had gender as major subject 5 years later.

Before doing so, it should be stressed that gender issues are not only related to the female sex. According to the definition, gender is ‘the state of being male OR female’. This means that one has to concentrate on those differences between males and females that need a different approach and/or a different treatment. Of course, there are typical sex-linked diseases such a breast cancer and prostate cancer. When narrowing to cardiology, there is for example peripartum cardiomyopathy and Duchenne cardiomyopathy [2–4]. More challenging from a scientific perspective is the potential disparity between men and women when it comes to diseases that affect both sexes, such as hypertension, coronary artery disease, atrial fibrillation, heart failure, and congenital heart disease [5–12].

Based on ACC CV News Digest over the period January till June 2015, there are some eye-catching examples. First an example of sex-related outcome of a common cardiovascular disease. On 16 May 2015, it was reported at the annual meeting of the Heart Rhythm Society that women who go through the menopause at a relatively young age [13] may have a slightly lower risk of developing atrial fibrillation. Nearly 18,000 middle-aged and older women in the USA were studied and it was found that women who had gone through menopause before the age of 44 were 17 % less likely to have atrial fibrillation. At the same meeting it was reported that safety levels of exercise differ between men and women with atrial fibrillation (second example). Data from 14 studies involving 380,000 people with atrial fibrillation showed that that moderate and vigorous levels of exercise are safe for women with atrial fibrillation but vigorous exercise may be hazardous for men.

A third example of a cardiovascular disease that occurs most commonly in postmenopausal women [14] was reported on 1 January 2015. It was found that hearts of patients with Takotsubo cardiomyopathy may not necessarily completely heal with time. Twenty-six patients with the disease underwent cardiac magnetic resonance 4 months after the acute event. It was shown that severely affected cardiac regions still showed wall motion abnormalities after 4 months. The findings were published in a letter to the editor of the Journal of the American College of Cardiology (JACC): Cardiovascular Imaging [15].

A fourth example regards isolated systolic hypertension. On 28 January 2015 it was reported that isolated systolic hypertension in young and middle-age adults is associated with an increased risk of death from cardiovascular causes later in life. There was a clear disparity in outcome between men and women. Males with a systolic blood pressure of 140 mmHg or higher and a diastolic blood pressure below 90 mmHg had a 28 % increased risk for death from coronary heart disease compared with men with normal blood pressure. However, women with isolated systolic hypertension had a more than 50 % greater death risk than women with optimal blood pressure. The findings were published in JACC [16].

The fifth example, presented at the ACC meeting in March this year, showed that women having an acute myocardial infarction (AMI) get to hospital later than men and are more likely to die. The study examined the records of 7457 European patients enrolled from 2010 to 2014 in the ISACS-TC registry and looked at in-hospital mortality, time delay to call emergency medical services (EMS), home-to-hospital delay using EMS, door-to-needle and door-to-balloon times and the overall time to treatment from symptom onset. It was found that 70 % of women took longer than an hour to get to a hospital whereas only 30 % of male patients with symptoms of AMI took that long. It was also found that women were nearly twice as likely as men to die in the hospital, 12 versus 6 %. Many delays occurred because women waited longer than men to call EMS.

Lastly, the sixth example, reported on 24 April 2015, shows that divorce is associated with an elevated risk of AMI, particularly in women. Women showed an independently significantly increased risk of an AMI whether they had been divorced once (by 24 %) or more than once (by 77 %), compared with continuously married women. In men, the risk elevation was only significant for those who had been divorced more than once (by 30 %), compared with continuously married men. Divorce is therefore a significant risk factor for AMI. The risks associated with multiple divorces are especially high in women and are not reduced with remarriage [17].

From these examples it is clear that gender disparities exist and continue to exist in cardiovascular disease. It is not our task to dissolve these differences but rather to identify them at an early stage, evaluate them thoroughly, and manage them accordingly [18].
